# Functional tumor-reactive CD8 + T cells in pancreatic cancer

**DOI:** 10.1186/s13046-025-03517-1

**Published:** 2025-08-25

**Authors:** Hongwei Sun, Changying Shi, Guoqing Fang, Qiufang Guo, Zhengliang Du, Geer Chen, Yasong Wu, Zhe-Sheng Chen, Jian Hua, Yan Zhang, Zhiwen Shi

**Affiliations:** 1 Zhejiang Provincial Key Laboratory of Medical Genetics, Key Laboratory of Laboratory Medicine, Ministry of Education, School of Laboratory Medicine and Life Sciences, Wenzhou Medical University, The First Affiliated Hospital of Wenzhou Medical University, WenZhou, China; 2 Shanghai JFKR Organoid Biotechnology Co., Ltd, Shanghai, China; 3 Hepatobiliary Surgery Center, Tongji Hospital, School of Medicine, Tongji University, Shanghai, China; 4 Shanghai Pharmaceutical Biotherapeutics, Cellular Therapeutics Center for Cancers, Co., Ltd, shanghai, China; 5 Chineo Medical Technology Co. Ltd, Beijing, China; 6 Department of Pharmaceutical Sciences, College of Pharmacy and Health Sciences, St. John’s University, Queens, NY USA

**Keywords:** Pancreatic cancer, Tumor-reactive CD8 + TILs, Deep learning, Single cell sequencing

## Abstract

**Background:**

Traditional methods for detecting tumor-reactive (TR) CD8 + tumor-infiltrating lymphocytes (TILs) in pancreatic cancer usually focus on neo-antigenic epitopes, which is limited by the narrow range of antigenic epitopes, and the lengthy and complex identification processes, resulting in an incomplete understanding of the biological characteristics of TR CD8 + TILs.

**Methods:**

This study introduces a novel approach that integrates single-cell sequencing with deep learning (DL), which enables the identification of tumor-reactive CD8 + T cells without neoantigen screening. The T Cell Receptor Engineered T (TCR-T) cell tumor organoid killing model was employed to validate the functionality of DL-identified TR CD8 + T cells, while spatial transcriptomics was used to confirm receptor-ligand interactions involving TR CD8 + TILs.

**Results:**

Comprehensive analyses of TR CD8 + TILs revealed impaired mitochondrial respiratory chain-related pathways regulated by the transcription factor FOS. The TIGIT-NECTIN2 axis was identified as an important immune checkpoint molecule in the tumor microenvironment of pancreatic cancer. T cell receptor (TCR) repertoire analysis demonstrated that some TR CD8 + TILs possess multiple TCR αβ combinations. Furthermore, TCR-T targeting experiments using tumor organoids revealed that combinations of multiple distinct TR TCRs exhibit significantly superior tumor-killing capabilities compared to a single type TCR. Clinically, a higher proportion of TR CD8 + TILs was positively associated with improved responses to neoadjuvant immunotherapy and longer overall survival in pancreatic cancer patients.

**Conclusion:**

This study represents a significant advancement in the understanding of TR TIL biology and provides a rapid and accurate method to identify TR CD8 TILs.

**Supplementary Information:**

The online version contains supplementary material available at 10.1186/s13046-025-03517-1.

## Introduction

Surgical resection is the most effective treatment modality for pancreatic cancer [1]. Unfortunately, only about 20% of newly diagnosed patients are eligible for surgical intervention. Furthermore, even among those who undergo surgery, up to 90% of patients face the challenge of disease recurrence. It is urgent to develop novel and effective treatment strategies for pancreatic cancer. Immunotherapy, particularly T cell-based approaches, has shown promise in enhancing antitumor immunity in pancreatic cancer s [2, 3]. Tumor-reactive (TR) T cells play a critical role in recognizing and eliminating malignant cells via major histocompatibility complex (MHC)/human leukocyte antigen (HLA)-restricted antigen presentation. Adoptive T cell therapies, including T cell receptor (TCR)-engineered T cells, have demonstrated efficacy in targeting tumor-specific or neoantigen-derived epitopes in pancreatic cancer [4]. However, identifying and characterizing TR T cells within the tumor microenvironment (TME) remains a major obstacle in advancing T cell-based therapies. Traditional ex vivo methods involve expanding tumor-infiltrating lymphocytes (TILs) and stimulating them with dendritic cells (DCs) pulsed with predicted neoantigen peptides [5]. While this strategy has been instrumental in detecting tumor-specific T cell responses, it has significant limitations. Firstly, identifying TR T cells through predicted somatic mutation epitopes overlooks those T cells in vivo that recognize other tumor antigen epitopes, such as cancer-testis antigens, tumor-associated antigens or neoantigens derived from aberrant RNA splicing [6, 7]. Secondly, many tumor-reactive TILs fail to expand ex vivo due to functional exhaustion, senescence, or an unfavorable TME, leading to missed detection of TR T cells [8]. Additionally, the entire identification process is high labor costs, time-consuming and requires complex platform, which has become a major factor limiting its clinical application.

**Fig. 1:**
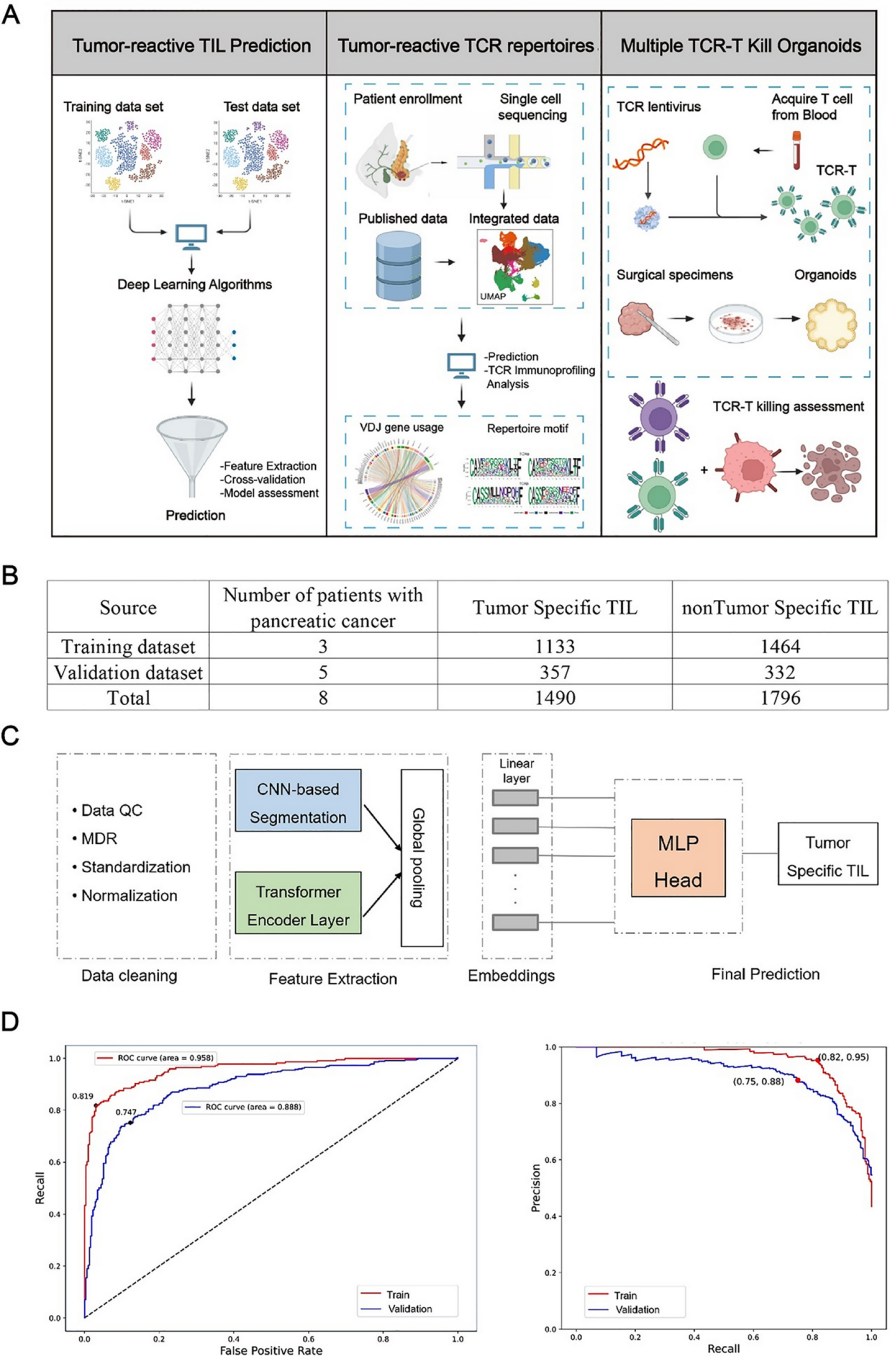
TR CD8 + T cells identification method using single-cell sequencing and deep learning algorithm. **A**: Workflow of data collection and analysis in this study. **B**: The model was trained using vast and diverse datasets, with an independent validation set of CD8 + TILs data points from three patients that the model had never encountered during training. **C**: The model architecture includes modules for data cleaning, pre-trained feature extraction, and embedding. MLP: Multilayer Perceptron; MDR: Missing Data Removal. QC: Quality Control. **D**: Receiver Operating Characteristic (ROC) curves were generated by training the model on a large dataset and testing it on an independent validation set (left panel). Precision-Recall (PR) curves were similarly generated by training the model on a large dataset and testing it on an independent validation set (right panel)

In recent years, tools or machine learning algorithms based on single-cell sequencing (scRNA-seq) for scoring gene sets have achieved efficacy in identifying tumor-reactive T cells [9, 10]. These methods perform well on training datasets, but the field continues to face challenges in making useful predictions on independent datasets. Some algorithms for identifying tumor-reactive (TR) cells do not distinguish between specific cancer types, which may lead to decreased sensitivity and specificity when applied to a particular cancer type [11]. Given the limitations of current methods for identifying TR CD8 + TILs, we leveraged prior experience in using scRNA-seq combined with machine learning for the identification of tumor neoantigen-specific T cells [12] to develop a deep learning (DL) approach for identifying TR CD8 + TILs in pancreatic cancer. The fundamental principle of this model is as follows: based on the information derived from single-cell TCR sequencing (scTCR) of postoperative tumor samples, the human T cell line was genetically modified by introducing mRNA gene constructs that encode TCRα/β pairs. These modified T cells were then cultured together with tumor cells derived from the same individual. T cell reactivity was assessed using flow cytometry analysis [13]. Using this method, both TR and non-TR TCR-T cells can be accurately identified and compared in a controlled laboratory setting. Additionally, we categorized CD8 + TILs based on the sequence data of their TCRs. The scRNA-seq data of these labeled CD8 + TILs was subsequently subjected to a transformer joint convolutional neural network (CNN) algorithm. This algorithm analyzed the matrices containing the expression profiles of individual cells and identified specific characteristics within them. Using this TR CD8 + TILs identification method, we can systematically investigate the distribution, biology, and composition of the TCR repertoire of TR CD8 + TILs (Fig. 1A). Our research demonstrates that the abundance of TR CD8 + TILs is a prognostic marker for neoadjuvant immunotherapy in pancreatic cancer, and that TCR-T with a combination of multiple TR TCRs has a significantly higher tumor-killing ability than a single type of TCR-T. Our work carries practical value in advancing the development of TCR-T therapies.

## Materials and methods

### Human samples

Three fresh specimens of pancreatic ductal adenocarcinoma and one pancreatic ductal squamous carcinoma were collected at the time of surgical resection.

### ScRNA and ScTCR sequencing

Single-cell suspensions were prepared using established protocols [14]. The cells were treated with FACS buffer, which is a solution of 1x PBS containing 1% BSA. PE anti-human CD8 antibodies (catalogue number: 344705, BioLegend) were used to stain the cells. The staining process lasted for 30 minutes at 4°C. After the staining procedure, the cells were washed and suspended again in FACS buffer. Afterwards, a subsequent staining with DAPI was performed for approximately 5 minutes. The cells that had been stained were subsequently separated using the CytoFLEX SRT flow cytometer, manufactured by Beckman. The Chromium Single Cell 5’ Gel Bead and Library Construction Kit (10× Genomics, #PN-1000006, PN-1000020) were used in combination with the Single Cell V(D)J Enrichment Kit Human T cell (10× Genomics, #PN-1000005) for scRNA-seq. The procedures of reverse transcription, cDNA recovery, cDNA amplification, and library construction were carried out following the instructions provided by the manufacturer. Afterwards, the libraries that were built were sequenced on the NovaSeq 6000 platform (Illumina) using paired-end sequencing and single indexing methodology.

### scRNA-seq and ScTCR data processing

Cells with fewer than 200 UMIs or more than 15% mitochondrial genes were excluded. Genes detected in more than three cells were kept. DoubletFinder was employed to detect and eliminate doublets from the dataset [15]. In cases where scRNA-seq data were directly obtained from CD8 + T cell sequencing and passed quality control, those cells were retained for subsequent analysis. Alternatively, a predefined set of signature genes (CD3D, CD3G, CD8A, CD8B) was utilized to identify CD8 + T cells. We merged CD8 + T cells obtained from various studies into a unified space using the fastMNN algorithm [16]. Subsequently, PCA, graph-based clustering, and UMAP were conducted using Seurat(V4) [14, 17]. The contig annotation outputs filtered by Cell Ranger VDJ were imported into R and analyzed using the scRepertoire (v1.1.3) R package [18]. Through the examination of public CDR3 TCR clonotypes [19] (https://vdjdb.cdr3.net/ ) that are responsive to common viral antigens such as influenza, CMV, SRAS, and EBV, we successfully identified a group of bystander T cells with the ability to recognize viruses.

### Plasmids and lentiviral transduction

HEK293T cells were transfected with pCDH-pTCR-Puro plasmid and viral packaging plasmids pMDLg/pRRE, pRSV-Rev, and pMD2.G using Opti-MEM reagent (Gibco). At 48 h post-transfection, lentiviral supernatants were collected, filtered through 0.45-microporous membrane filters, concentrated by ultracentrifugation and frozen in aliquots at 80℃ prior to use.

### Organoid culture

Pancreatic ductal adenocarcinoma tumor tissues are processed, first by washing twice with PBS. The tissues are then minced in a petri dish with shears into 1–3 mm³ fragments or smaller. The fragments are transferred to a 15mL Falcon tube with 5mL of tumor tissue digestion solution. Then tissue fragments are incubated in the solution at 37 °C for 15–20 min and intermittently mixed every 5 min. Digestion is evaluated with light microscopy for dissociated fragments. Once at least 80% single cells are present in the mixture, PBS is added, and the mixture is filtered using a 100 μm cell strainer. The supernatant is transferred to a separate 15 mL Falcon tube and centrifuged at 300 x g for 5 min at 4 °C. Next, the pellet is resuspended evenly in a 70:30 mixture of Matrigel^®^ Matrix (Corning, Catalog 356231) and organoid media, and placed on ice to prevent polymerization prior to plating. The mixture is plated into 50 µL domes on a prewarmed 24-well plate (Corning, Catalog 3524). The plate is placed in a CO₂ incubator (37 °C, 5% CO₂) for 10 min to allow the Matrigel to polymerize and solidify, after which 0.75 mL of endometrial cancer organoid culture medium (JFKR, Catalog JFKR-PADC-100, Shanghai, China) is added to the tumor endometrial organoid wells. Culture Medium was changed every 2–3 days and after 10 days of culture, organoids were harvested for further processing. Human Pancreatic-ductal-adenocarcinoma cancer organoid culture medium (JFKR, Catalog.FKR-PADC-100) contain: Advanced DMEM-F12(Gibco, cat. 12634-028),50ng/L Wnt3A (Peprotech,315-20-2UG), 1% Glutamax (Life Technologies, Catalog. 35050061), 1% HEPES ((Gibco, cat. 15630-056), 1% penicillin-streptomycin (Gibco, cat. 15070063), 2% B27 (Gibco, cat. 17504-044), 1% N2 (Gibco, cat. 17502048),500 ng/mL human R-Spondin 1 (JFKR, Cat, JFKR-RP01-100ug), 0.2% Primocin (Gibco, cat. PML-40-60), 50 ng/mL human EGF (JFKR, Cat. JFKR-RP03-100ug), 100 ng/mL human FGF10 (JFKR, cat. JFKR-RP04-100ug), 1.25 mM N-acetyl-L-cysteine (Sigma-Aldrich, cat. A9165-5G), 1 mM nicotinamide (Sigma-Aldrich, cat. N0636) ,0.5 mM A83-01 (Tocris, cat. 2939). Organoids were generated, passaged for 3–4 generations, and then cryopreserved. The recovered organoids used in our study were between the 5th and 7th generations, and the culture medium was changed every three days. Cell number and viability were assessed using NucleoCounter AO/PI staining (ChemoMetec, Catalog NC-200 NucleoCounter) on living organoids. The stained organoids were analyzed using the IncuCyte^®^ Live-Cell Analysis S3 system (Sartorius, Catalog Incucyte S3).

### T cell transfection and Co-culture with Organoid

PBMCs from patients were cultured in T-Cell Expansion Basal Medium (Gibco, cat. A1048503) with 100 U/ml IL-2(Peprotech, cat. 200-02-50UG), 3ug/ml Anti-human CD3/CD28 Dynabeads (Gibco, cat. 11161D). Lentiviral transduction of T cells was carried out with TCRs incorporating murine-derived constant regions. Transfection efficiency was measured by the expression of a marker using flow cytometry on day 2 and day 8 after transduction. Transduced T cells were cultured with replaced fresh medium and IL-2 every 3 days, and were cryopreserved in 10 days post transduction. Pancreatic ductal adenocarcinoma cancer organoids were partially digested to remove Matrigel while retaining the three-dimensional architecture. The remaining Matrigel was washed away before coculture. Organoids were mechanically broken and transferred into each well of a 24-well plate, containing 250 µl of T cell medium. T cells were harvested, counted, and combined with organoids at a ratio of 25–50:1 (T cells to organoids). Co-culture was performed with T cells for 48 h in a 37 °C, 5% CO₂ incubator (Sartorius, Catalog Incucyte S3).

### JC-1 mitochondrial membrane potential assay

After collection, T cells were washed twice with DPBS supplemented with 2% FBS and stained with JC-1 dye at a final concentration of 2 µM for 20 min at 37 °C in the dark. Following incubation, cells were washed twice with DPBS and resuspended in 500 µL of DPBS supplemented with 2% FBS. Fluorescence signals were acquired using a CytoFLEX flow cytometer (Beckman Coulter) to measure red (585 nm) and green (525 nm) fluorescence.

### Flow cytometry

After collection, cells were washed twice with staining buffer (DPBS supplemented with 2% FBS) and treated with Fc-Block (BD Biosciences) for 10 min at room temperature (RT) and then stained with the anti-mouse TCR βchain antibody(Biolegend,109212) for 30 min at RT, then washed with staining buffer, resuspended with 500 uL staining buffer and collected on CytoFLEX (Beckman Coulter). Analysis was performed using FlowJo software.

### Trajectory and simultaneous gene regulatory network analysis

The developmental trajectory of the indicated cells was inferred through the use of the Monocle3 package (http://cole-trapnell-lab.github.io/monocle-release/monocle3 ) and developmental pseudo-time analysis. First, the Seurat object’s raw count was converted to a CellDataSet object. The ordered genes that were most likely to be useful for ordering cells along the pseudotime trajectory were chosen using the clusterData() function. The RcisTarget and GRNBoost motif databases were used, along with the default parameters for the pySCENIC [20] analysis. Using the RcisTarget package, we found transcription factor-binding motifs that were overrepresented on a gene list. The AUCell package was used to score each regulon group’s activity for each type of cell. The limma [21] package was applied to analyze differential regulators between various groups.

### Cell-cell communication analysis with cellchat

Utilizing the CellChat [22] R package, potential intercellular communication was evaluated. The CellChat() function was used to create a CellChat object by importing the normalized expression matrix. Then, using default parameters, the data were preprocessed using the identifyOverExpressedGenes(), identifyOverExpressedInteraction(), and ProjectData() functions. Next, potential ligand-receptor interactions were found using the computeCommunProb(), filterCommunication(), and computeCommunProbPathway() functions. Lastly, the aggregateNet() function was used to aggregate the cell communication network.

### Multiplex immunohistochemistry

A standardized protocol was employed to perform four-color multiplex immunohistochemistry. Tumor sections of 4–5 micrometers were obtained from formalin-fixed paraffin-embedded pancreatic cancer specimens. They were deparaffinized, hydrated, and manually stained with antibodies against CD8 (GB12068, Servicebio), NECTIN2 (NBP1-91211, NOVUS), TNFSF10 (MAB375, NOVUS), and PANCK (GB122053, Servicebio). Tyramide signal amplification (TSA) with different wavelength-emitting dyes was used. The stained slides were then imaged and scanned using the Pannoramic MIDI imaging system.

### Dataset collection

10x genomic scRNA-seq data from TR CD8 + T cells identified by verification experiment of co-cultivation between TCR-T cells and tumor PDX models were a generous gift from Dr Zibo Meng and Prof Rienk Offringa. A total of 3,286 experimentally verified CD8 + T cell single-cell sequencing data was used to build and validate the DL model. The published pancreatic cancer tumor TIL scRNA-seq data could be found in Table S1 [13, 23–29]. Pancreatic cancer bulk RNA-seq cohort data analysis from GEPIA [30].

### Spatial transcriptome analysis

Normalized expression data from each spatial feature were analyzed using ESTIMATE [31], which provided stromal scores, immune scores, and tumor purity metrics for each feature. At the feature level, single sample GSEA (ssGSEA) [32] was applied to expression data from each spatial feature, using gene sets from the Hallmarks collection.

### Deep learning model generation

We used the R package SCTransform to re-normalize the single cell sequencing data sets [33]. The top 10,000 genes were identified as highly variable genes (HGVs). The HGVs are then further filtered using two filter conditions: (a) screening for intersecting genes in HGVs from different datasets; and (b) filtering out genes on the gene blacklist (Table S2). The gene blacklist includes T cell receptor (TCR) and disassociation-induced genes. We then calculated the z-score for the scale data. We use a random forest model-based approach to assess the feature importance of HGVs and filter out feature variables with weight coefficients greater than 0.0025 before establishing a DL model (Table S2) [34].

The DL approach incorporates a Transformer encoder layer with residual connections and a regression model that comprises convolutional layers, a series of Transformer encoder layers, and fully connected layers. During the training process, the model undergoes forward and backwards propagation to learn from the training data. The model parameters are updated using a suitable loss function and optimizer. Evaluation involves the assessment of model performance on the test dataset, usually by calculating loss or other metrics that compare predicted outputs to true labels to measure accuracy and generalization. The model is trained for a specific number of epochs using the given data loaders. During training, both training and testing losses are computed to monitor performance.

### Chip-seq analysis

Chip-seq data were obtained from the Gene Expression Omnibus under accession no. GSE116698/ GSM935355 [35]. Data analysis was performed as previous described [36]. Briefly, 75-bp reads were aligned to the human genome (hg19) using Bowtie2. For peak calling, MACS2 software was used [37]. The visualization part of the chip-seq analysis is done in deeptools and IGV software [38, 39].

### Analysis of TR CD8 + T cells composition in the PDAC neoadjuvant immunotherapy cohort

To assess the enrichment of TR CD8 T cells within CD8 + TILs from PDAC patients, we applied the BayesPrism [40] algorithm to deconvolute transcriptomic data from the neoadjuvant therapy cohort. To minimize batch effects and enhance computational efficiency, the analysis was restricted to protein-coding genes. We employed the default settings for Gibbs sampling and optimization. The final cell type fraction for each bulk RNA-seq sample was derived from the refined theta matrix and subsequently utilized for downstream analyses.

### Statistical analysis

For comparisons between two groups, Welch’s t-test was chosen for group comparisons due to unequal variance across conditions. For tumor versus normal tissues, non-parametric Mann–Whitney U test was used. Clinical data were analyzed using univariate Cox proportional hazards regression to evaluate the association between individual clinical variables and patient survival outcomes. Hazard ratios (HRs), 95% confidence intervals (CIs), and corresponding P-values were calculated to assess statistical significance. The cut-off values for stratifying patients in Figs. 9B and D were determined using the best-cut off method [41]. All statistical analyses were conducted using R (version 4.2) or GraphPad Prism (version 9), and *P*-values < 0.05 were considered statistically significant.

## Results

### DL approach for identifying TR CD8 + T cells

We systematically gathered datasets comprising currently available TR CD8 + TILs in pancreatic cancer for DL approach construction, accumulating over 3200 scRNA-seq data of TR CD8 + TILs (Fig. 1B). To construct the DL approach, we employed a staged method, dividing the task of learning the characteristics of TR CD8 + TILs into three steps to reduce the complexity of the prediction tasks (Fig. 1C). Initially, we mitigated the impact of technical variability by employing the SCTransform algorithm. Subsequently, we standardized the single-cell expression matrix for each sample using the z-score method, ensuring that data from various samples remained unaffected by differences in sequencing depth and batch effects. Following this, we conducted annotation of the subpopulations of CD8 + TILs. These three steps produced numeric and categorical vectors that are amenable to mathematical operations, setting the stage for the final pairing prediction. We simultaneously tested various deep learning architectures and ultimately selected the Transformer joint 1D-CNN model. This model effectively utilizes embeddings to incorporate meaningful insights from the sequencing matrix and categorical vectors in a biologically relevant manner. We applied fine-tuning techniques to enhance the accuracy of the predictive model in detecting TR CD8 + TILs.

The evaluation of our DL approach primarily relies on two metrics: the area under the curve (AUC) of the receiver operating characteristic (ROC) and the precision-recall (PR) curve. Notably, the AUC achieved a value of 0.958 for the ROC and 0.95 for the PR curve in the training dataset (Fig. 1D, left panel). To verify the accuracy of the model’s predictions, we tested it using a separate set of scRNA-seq data that had been previously validated by CD8 + TILs targeted tumor killing assays. The model’s prediction consistently demonstrated high performance, with an accuracy of 0.888, a precision of 0.7916, and a recall of 0.88. Collectively, these findings suggest that our DL approach demonstrates strong performance in detecting TR CD8 + TILs in pancreatic cancer.

### TCR-T targeted killing on organoids validated the tumor-reactive TCRs identified by the DL approach

**Fig. 2:**
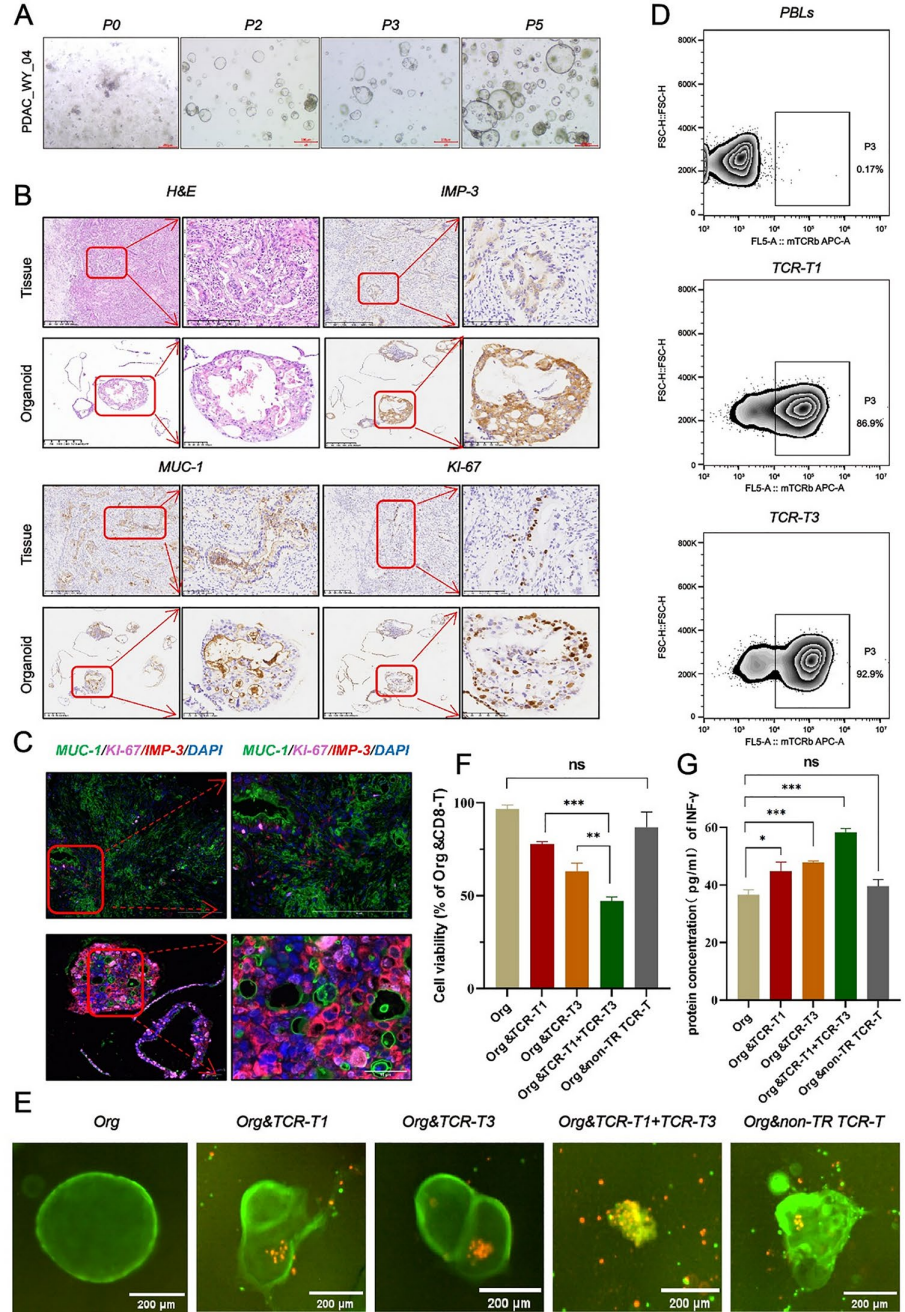
TCR-T Cell-Mediated Cytotoxicity Against Tumor Organoids. **A**: Bright-field microscopy images showing the growth and morphological changes of patient-derived tumor organoids (WY_04) at different passages (P0, P2, P3, and P5). **B**: Immunohistochemical analysis of tumor tissues and organoids, demonstrating the expression of key tumor markers. **C**: Multi-plex immunofluorescence analysis of tumor organoids to detect the expression of specific tumor markers. **D**: Flow cytometric analysis showing the expression positivity rate of the exogenous TCR gene in autologous peripheral blood lymphocytes (PBLs) from the patient, 8 days post-transfection. **E**: Representative confocal microscopy images of organoids co-cultured with TCR-T cells, stained with acridine orange/propidium iodide (AO/PI) to assess cell viability and cytotoxicity. Org: organoids. non-TR TCR-T control: Non-tumor-reactive TCR control group. **F**: The statistical results of the number of organoids, compared with the control group (Org& non-TR TCR), indicated significant differences (Welch’s t-test : ** *P* < 0.01; *** *P* < 0.001). **G**: IFN-γ ELISA assays of coculture of TCR-T cells and organoids (Welch’s t-test: *** *P* < 0.001)

To validate the specificity of the TCRs identified by this algorithm, we included a patient with PDAC in the study. Postoperative tumor sample from this patient were used to establish organoid cultures. As shown in Fig. 2A and B, we successfully constructed organoids derived from the patient’s tumor samples, which expressed tumor-specific markers MUC-1 and IMP-3, as well as the proliferation marker KI-67 (Fig. 2C). Through scRNA-seq and scTCR-seq of TILs, we identified two TR TCRs (which were synthesized in vitro with murine substitutions in their constant regions [42]). Flow cytometry analysis confirmed the proper expression of both TCRs (Fig. 2D). Subsequently, an organoid T cell co-culture system was employed to evaluate the tumor-killing efficiency of the identified TCR-T cells (#1 and #3). Both TCR-T cells recognized and attacked autologous organoids, significantly affecting the survival of the organoid cells. Live imaging with Acridine Orange/Propidium Iodide (AO/PI) staining showed a marked increase in cell death in autologous organoids co-cultured with TCR-T cells compared to those co-cultured with non-transduced CD8 + T cells or non-TR TCR-T control cells (Figs. 2E and F). An Interferon-gamma (IFN-γ) enzyme-linked Immunosorbent Assay (ELISA) further demonstrated that TCR-T cells secreted high levels of IFN-γ (Figs. 2G). Additionally, our experimental results indicated that a combination of multiple TR TCRs exhibited enhanced targeting and killing effects on the organoids (Figs. 2E, F and G).

### A high-resolution cellular landscape of TR CD8 + TILs in pancreatic cancer

**Fig. 3:**
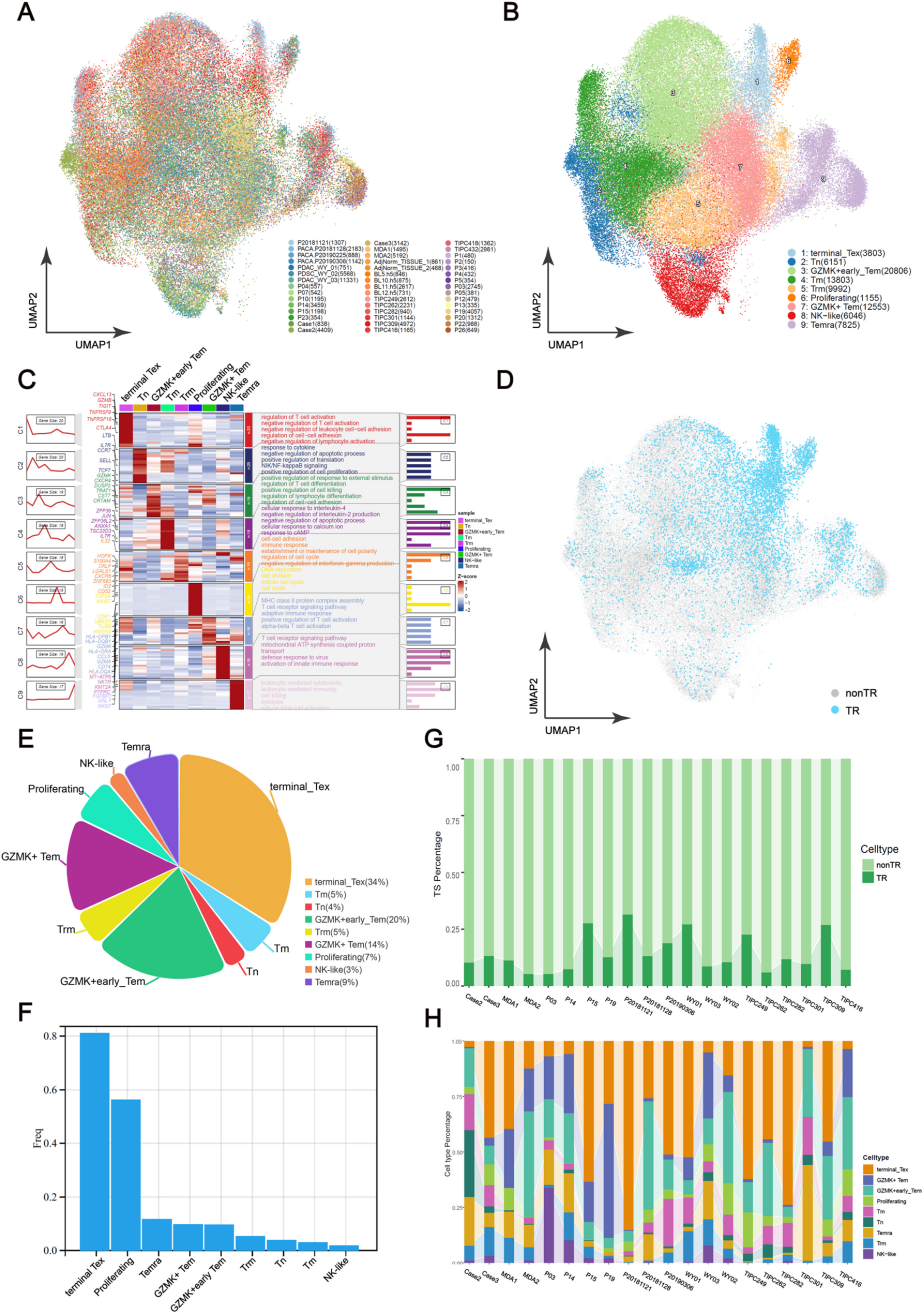
Distribution characteristics of TR CD8 + T cells in pancreatic cancer patients. (**A** and **B**) UMAP plot of CD8 + TILs from 41 pancreatic cancer patients. Tem: effector memory cells; Temra: terminally differentiated effector memory cells; Tex: terminally exhausted T cells; Trm: tissue-resident memory T cells; NK-like: natural killer cell-like, Tn: innate T cells, Tm: memory T cells. (**C**) Cluster plot showing the expression of marker genes and pathway enrichment analysis of the nine types of CD8 + TILs (Table S6). (**D**) UMAP plot of all TR CD8 + TILs from all patients. TR: Tumor Reactive. (**E**) Pie chart representing the proportion of TR CD8 + T cells in different subtypes of T cells. (**F**) Boxplot representing the abundance of TR CD8 + T cells in different subtypes of T cells. (**G**) The bar chart depicts the proportion of TR CD8 + T cells in CD8 + TILs in different patients. (**H**) The bar chart depicts the subtype distribution of TR CD8 + T cells

We assembled the largest single-cell transcriptome atlas of CD8 + TILs in human pancreatic cancer to date, enabling a comprehensive investigation of the biological properties of TR CD8 + TILs. Rigorous quality control filtering was applied to a dataset comprising 82,134 CD8 + T cells. These cells were derived from three newly generated samples and scRNA-seq data from 38 published pancreatic cancer patients. Thirteen distinct clusters with unique gene signatures were identified after removing batch effects and performing unsupervised clustering on data from various sample sources (Fig. 3A and Figure S1A and B). Using the FindMarkers algorithm and a cell marker database, we identified nine T cell subtypes [23], including GZMK + effector memory cells (GZMK + Tem/early Tem), terminally differentiated effector memory cells (Temra), terminally exhausted T cells (Tex), tissue-resident memory T cells (Trm), natural killer cell-like (NK-like) T cells, innate T cells (Tn), memory T cells (Tm), and proliferative T cells(Fig. 3B). The expression of characteristic genes and enriched signaling pathways varies significantly across these T cell subsets (Fig. 3C and Figure S1C).

Subsequently, we employed the DL approach to distinguish TR CD8 + TILs from non-TR CD8 + TILs and mapped them onto the UMAP clustering plot (Fig. 3D). This revealed a distinct clustering pattern for the TR CD8 + T cells. Specifically, 34% of TR TILs were found in the terminally exhausted T cell subset. Additionally, more than one-third of TR CD8 + TILs were present in the GZMK + Tem subset, with the remaining cells distributed among the Temra and proliferative T cell subsets (Fig. 3E). Moreover, we discovered that TR T cells constituted up to 80% of the overall exhausted T cell population and over 50% of the proliferative T cell population (Fig. 3F). In contrast, virus-specific T cells displayed a more widespread distribution compared to the concentrated distribution of TR CD8 + T cells, with notable enrichment in the memory T cell subset (Figure S1D and E).

The abundance of TR CD8 + T cells among CD8 + TILs varies in pancreatic cancer patients, ranging from 4.92 to 36.6% (Fig. 3G). Additionally, there are significant variations in the distribution of TR TILs subtypes among different patients. For instance, in patients P20181121, TIPC249, and TIPC282 (with the majority of TR TILs in TIPC249 and TIPC282 validated through the PDX model), TR TILs are primarily concentrated in the terminally exhausted T cell subset. In contrast, in patients P20181128, WY02, and TIPC309 (the majority of TR CD8 + T cells in patients TIPC309 have been validated through PDX model), there is a high proportion of TR TILs in the GZMK + early Tem subtype (Fig. 3H).

### The expression of genes related to mitochondrial respiratory chain pathway is significantly downregulated in TR CD8 + T cells

**Fig. 4:**
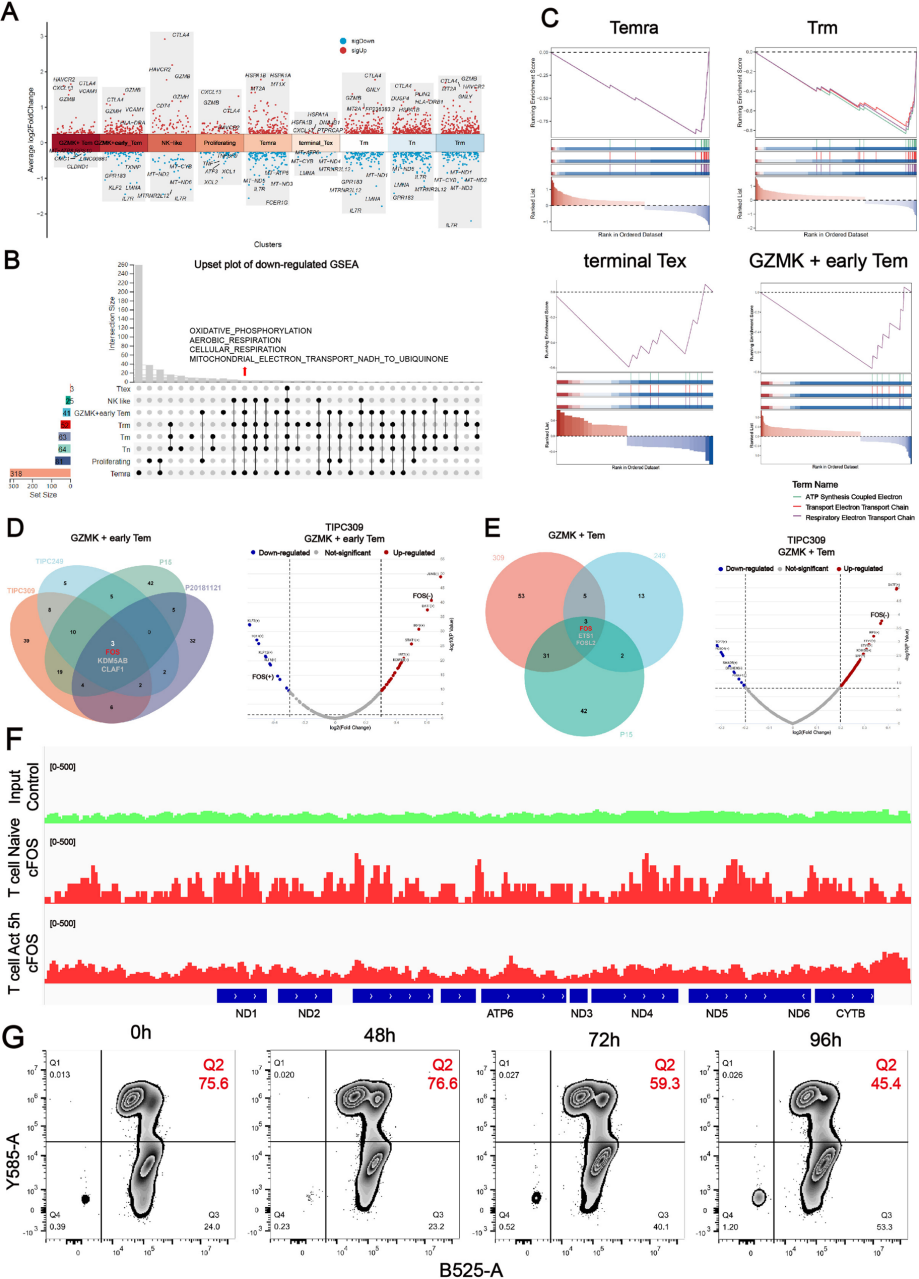
Biological characteristics of TR CD8^+^ T cells in human pancreatic cancer. (**A**) DEGs analysis showing up- and down-regulated genes across different subtypes of TR CD8 + T cells. DEG: differentially expressed genes. (**B**) Upset plot showing the overlap of GO pathways between the indicated comparisons of the different subtypes of TR CD8 + T cells. (**C**) Enrichment plot for the set of DEGs in the transcriptome of TR CD8 + T cells versus Non-TR T cells in the Exhausted and Tem types of T cells by GSEA; *p* values were determined by a one-tailed permutation test by scGSEA. NES, normalized enrichment score. (**D** and **E**) The Venn plot represents the enriched FOS regulons through regulon analysis in the GZMK + Tem subtype of TR CD8 + T cells from four patients (TIPC249/309, P15 and P20181121). The volcano plot showing the significantly upregulated FOS (-) regulon and the significantly downregulated FOS (+) regulon in TR CD8 + T cells of the TIPC309 patient. (**F**) The Integrative Genomics Viewer screenshots show open chromatin at the mitochondrial loci in naive and activated T cells at 5 h. The y axis shows Chip-seq coverage by estimated fragments normalized to the number of mapped reads. (**G**) JC-1 mitochondrial membrane potential assay showing signal intensity at different time points during continuous activation of T cells

The widespread identification of TR CD8 + T cells facilitates further investigation into their biological properties. The differentially expressed genes (DEGs) between TR CD8 + T cells and non-TR CD8 + T cell subsets were compared. Our differential analysis revealed that TR CD8 + T cells from various subsets exhibit elevated expression of T cell exhaustion-related genes, such as *CTLA4*, *CXCL13*, and *HAVCR2*, which is consistent with a previous research [43] (Fig. 4A and Figure S2B). One particularly intriguing discovery is that TR CD8 + T cells across various subsets commonly downregulate genes associated with the mitochondrial respiratory chain pathway, such as *MT-ATP6*, *MT-ND1*, and *MT-ND3* (Figure S2A and C). We then investigated the biological mechanisms differentiating TR CD8 + T cells from non-TR CD8 + T cells using these DEGs in a single-cell gene set enrichment analysis (scGSEA). The upset plot demonstrated that different TR CD8 + T cell subtypes had enriched mitochondrial respiratory chain pathways. Signal intensity levels for the ATP synthesis-coupled electron transport, electron transport chain, and respiratory electron transport chain pathways were markedly diminished in all TR CD8 + T cell subtypes, except proliferating T cells (Figs. 4B, C and Figure S2A, C). Through analysis of regulons in GZMK + Tem subtype TR CD8 + T cells from four patients (TR CD8 + T cells of TIPC309/249 were verified by T cell-targeted killing experiments), we discovered that all patients have enriched FOS regulons (Figs. 4D, E and Figure S2D). The FOS (-) regulon is significantly upregulated in TR CD8 + T cells, while the FOS (+) regulon is significantly downregulated, as shown by the volcano plot. The FOS transcription factor plays a significant role in regulating the expression of genes associated with the mitochondrial respiratory chain pathway (https://guolab.wchscu.cn/hTFtarget/#!/). To confirm the function of FOS in T cells, human naive T cells isolated from healthy donors’ blood were activated with anti-CD3/CD28 beads for 0 and 5 h before performing ChIP-seq for cFOS. The ChIP-seq analysis results show that, compared to the case group, naive T cells exhibit open chromatin formation in mitochondria upon activation. As T cells continue to be activated, mitochondrial open chromatin formation weakens (Fig. 4F). JC-1 assay results demonstrated that the proportion of T cells with high membrane potential decreased from over 70–45% after more than 72 h of continuous activation (Fig. 4G).

### The pseudo-time trajectory of TR CD8 + T cells

**Fig. 5:**
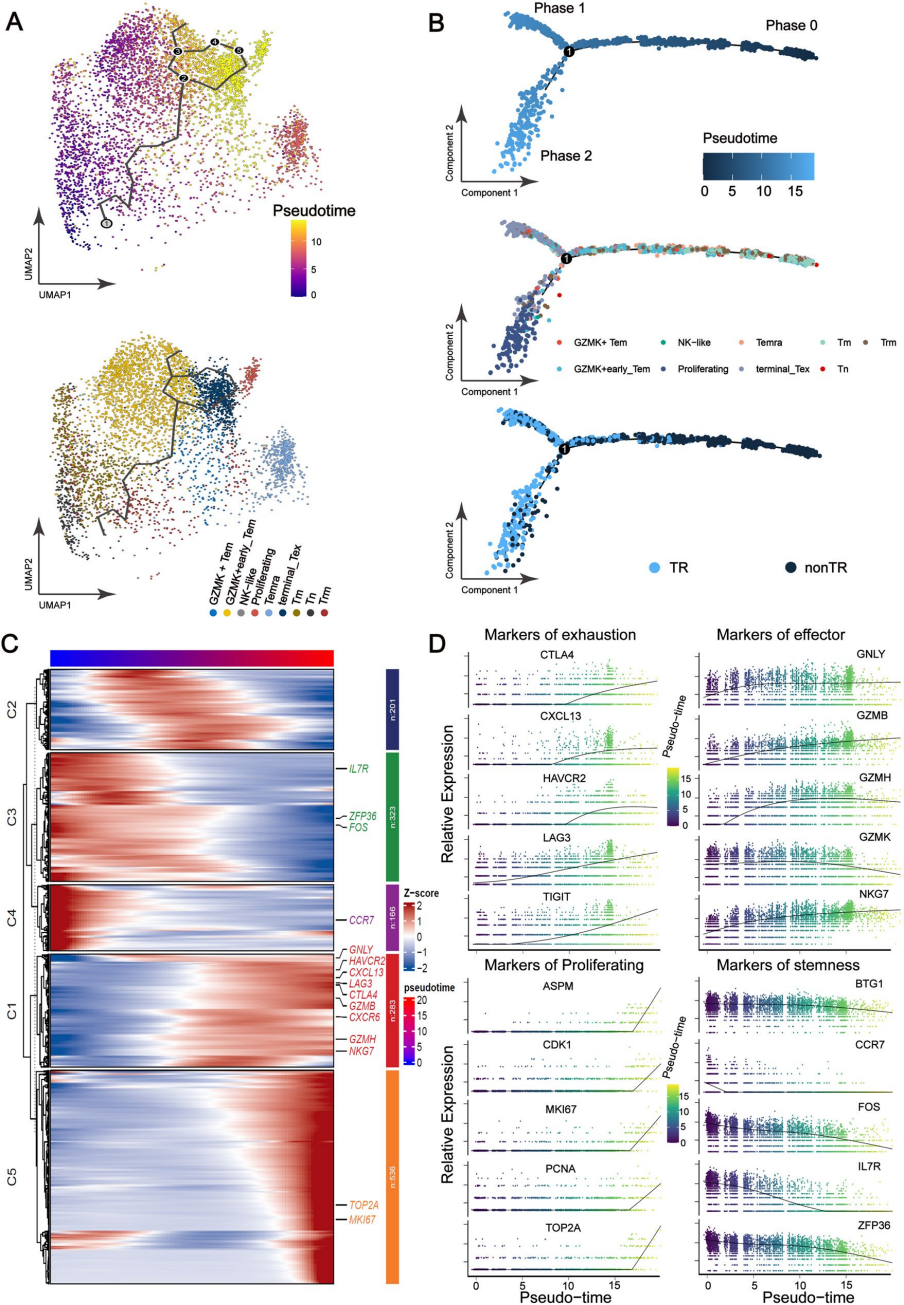
The pseudo-time trajectory of TR CD8 + TILs. (**A**) Pseudo-time trajectory of these TR CD8 + T cells identified two distinct cell fates, colored by pseudo-time or CD8 + T cell subtypes. The black line and numbers indicate the potential evolutionary direction in the trajectory. (**B**) Pseudotime-ordered analysis of CD8 + TILs identified two distinct cell fates, colored by subclusters. (**C**) Heatmap displaying the dynamic changes in gene expression along the pseudo-time. Pseudo-time subclusters are labeled by colors. (**D**) Two-dimensional plots showing the dynamic expression of selected genes along the pseudo-time, colored by subclusters

Next, we illustrated the pseudo-time trajectory of these TR CD8 + T cell subtypes. On a global scale, the diffusion map showed that CD8 + T cells develop from naïve T cells to either terminally exhausted (Tex) or proliferating T cells (Fig. 5A). In phase 0, the Tn and Tm subsets were the dominant groups (Fig. 8B and Figure S2E) and exhibited elevated expression of genes associated with naïve or memory T cells, such as *FOS*, *CCR7*, and *IL7R* (Figs. 5C, D and Figure S2F). TR CD8 + T cells undergo a differentiation process within the pseudotime range of 5–10, primarily giving rise to the Temra subset. Following this stage, they diverge into two distinct cellular fates. Phase 1 consisted mainly of the terminally exhausted T cell subset, followed by the GZMK + Tem subset (Fig. 5B), both of which exhibited high levels of effector-related or exhaustion markers (Fig. 5D). Phase 2 was primarily composed of the proliferating T cell subset, which exhibited high levels of proliferation-related molecules (Figs. 5B, D and Figure S2G).

### Ligand-receptor analysis revealed that the NECTIN2-TIGIT interactome was enriched in TR CD8 + T cells

**Fig. 6:**
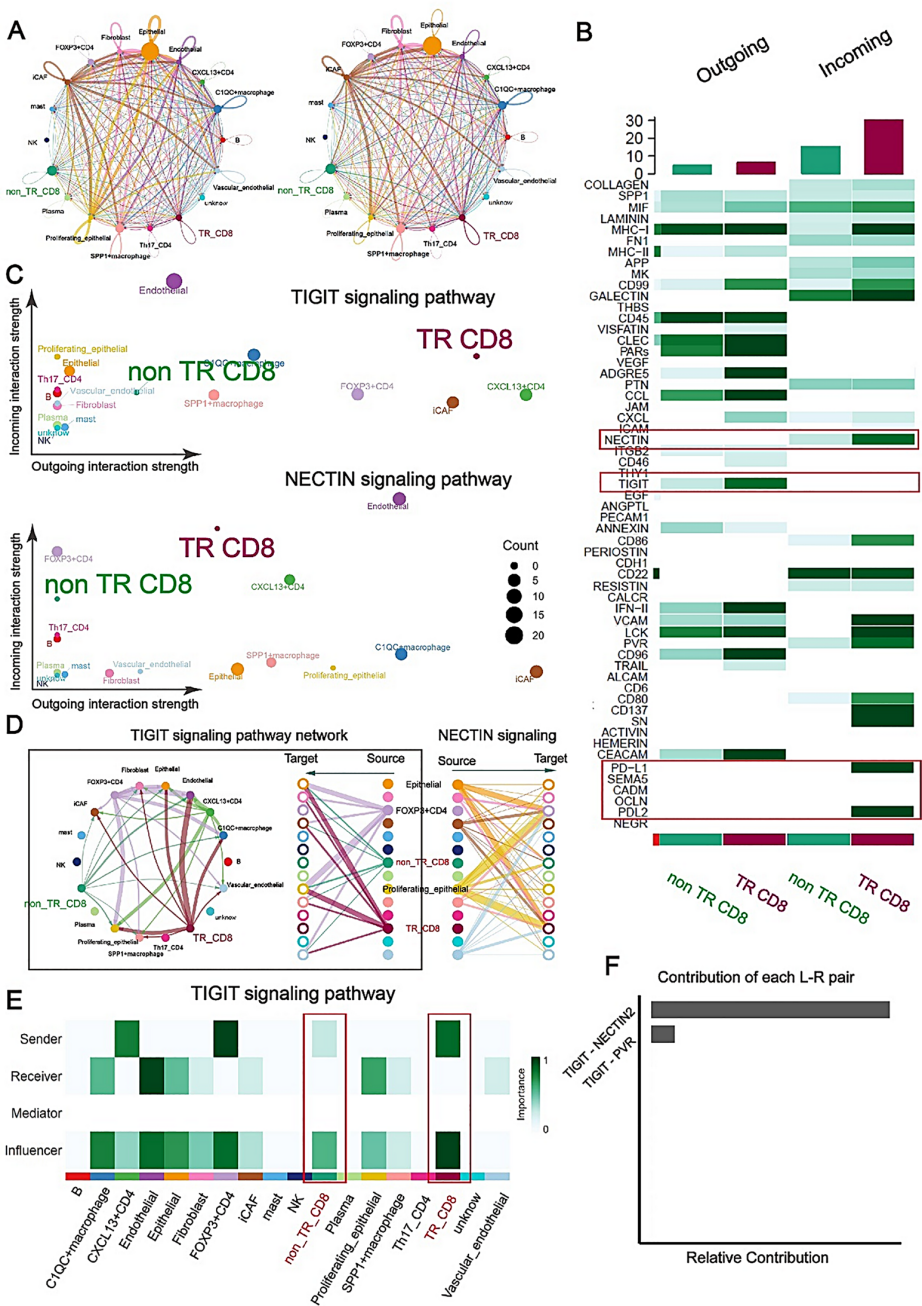
Ligand–receptor analysis reveals TR CD8 + TILs interactomes. (**A**) Intercellular communication networks showing the number and strength of the 18 annotated cell subtypes. (**B**) The heatmap plot illustrating the significantly different incoming and outgoing pathways between TR and non-TR CD8 + TILs. (**C**) The scatter plot depicts the incoming and outgoing interaction strength of the 18 annotated cell subtypes in TIGIT and NECTIN signals. (**D**) Intercellular communication networks showing the strength of cell subtypes in TIGIT and NECTIN signals. (**E**) The heatmap plot illustrating the significantly different incoming and outgoing TIGIT pathway between TR and non-TR CD8 + TILs. (**F**) The box plot illustrates the relative contributions of various ligand-receptor (L-R) pairs in NECTIN signaling pathway

The intercellular communication detected by CellChat analysis is extensive and complex (Fig. 6A). TR CD8 + T cells exhibited greater cellular communication than non-TR CD8 + T cells, particularly through exhaustion-associated receptor-ligand interactions, as reflected by their higher incoming and outgoing interaction strengths (Fig. 6B and Figure S3A). We observed that the TIGIT and NECTIN signaling pathways were more intense in TR CD8 + T cells compared to non-TR CD8 + T cells (Fig. 6C). In TIGIT signaling, TR CD8 + T cells primarily serve as initiating entities, exhibiting a pronounced outward signaling tendency. Conversely, in NECTIN signaling, epithelial and proliferative epithelial cells display a strong propensity for outward signaling, with TR CD8 + T cells predominantly acting as the main signal-receiving entities (Fig. 6D and E). Analysis of ligand-receptor interactions revealed activated signals between *TIGIT* and *NECTIN2* in TR CD8 + T cells and epithelial cells (Fig. 6F). Additionally, we observed significant enrichment of signaling pathway interactions, including CD99, IFN-II, and CD80, in tumor-reactive (TR) CD8 + T cells (Figure S3B-D). A unique TNFSF10-TNFRSF10B interaction was observed between TR CD8 + T cells and epithelial cells (Figure S3E and F).

### The Spatial transcriptome revealed the expression patterns of NECTIN2 within the tumor microenvironment

**Fig. 7:**
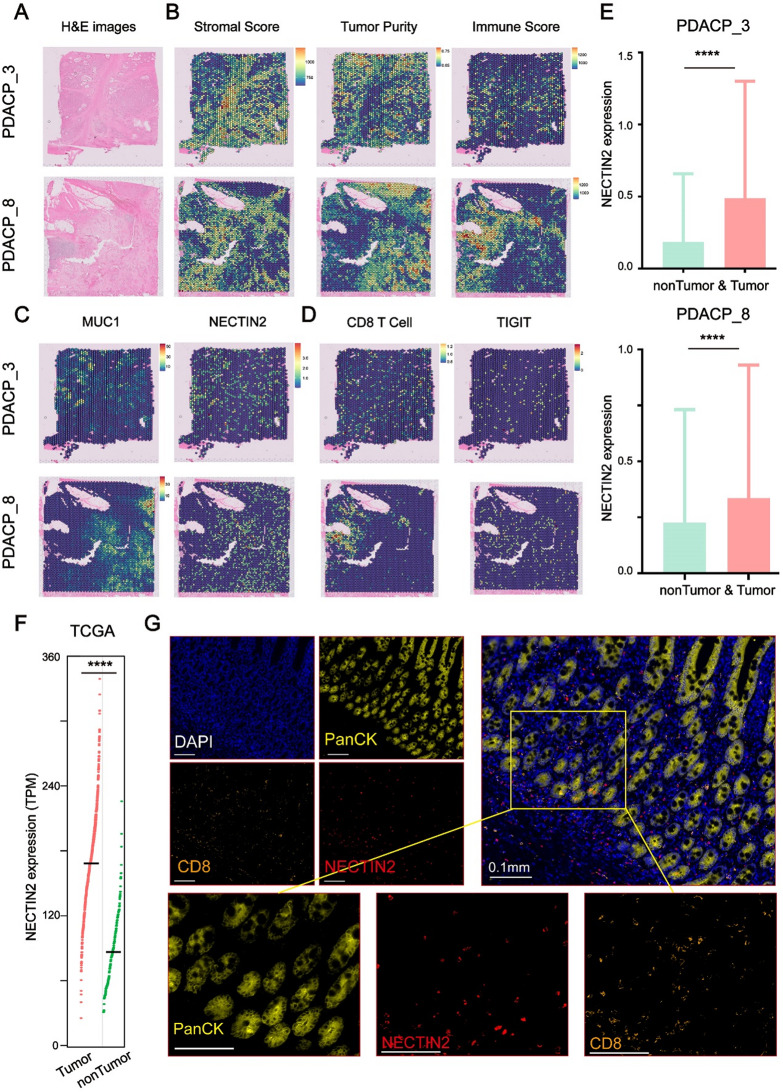
The spatial transcriptome reveals the expression patterns of NECTIN2. (**A**) H&E-stained images of sample PDACP_3 and PDACP_8. (**B**) Expression data per spatial feature was analyzed using ESTIMATE to generate a tumor purity score, stromal score, and immune score. (**C**) Spatial plots of MUC1 (Tumor marker) and NECTIN2. (**D**) Per-feature enrichment scores for CD8 T cell signature as determined by ssGSEA and Spatial plots of TIGIT. (**E**) The box plot showed the expression levels of NECTIN2 in tumor and non-tumor areas in the spatial transcriptome (Welch’s t-test, ****: *P* < 0.0001). (**F**) The box plot showed the expression levels of NECTIN2 in tumor and non-tumor tissues in TCGA-PDAC cohort (Mann–Whitney U test, ****: *P* < 0.0001). TCGA: The Cancer Genome Atlas. (**G**) Tumor sections from patients with pancreatic cancer stained with anti-PanCK (yellow), anti-NECTIN2 (red), anti-CD8(orange) and DAPI

To gain deeper insights into the spatial interactions between NECTIN2 and TIGIT, we analyzed two representative tissue samples manually annotated by a board-certified anatomic pathologist: (1) a sample characterized by tumor and fibrosis-dominant regions (PDACP_3) and (2) a sample exhibiting a dense ring of lymphoid cells (PDACP_8) [44]. Normalized gene expression data from these samples were subjected to ESTIMATE analysis to delineate their transcriptional substructures. The results from ESTIMATE closely aligned with manual histological annotations and were corroborated by the expression of tumor-associated marker genes (Figs. 7A-C). To further characterize the infiltrating immune cells, we employed a set of CD8 T-cell-specific expression signatures [45]. Spatial analysis revealed that regions enriched for T cells corresponded to the dense lymphoid ring observed in PDACP_8 (Figs. 7A and D). By analyzing the spatial transcriptome data of two representative tissue pathology samples, we found that NECTIN2 was mainly expressed in tumor tissues, which was consistent with the sequencing results of bulk RNA-seq (Fig. 7C, E, F and Figure S3G). To validate the spatial expression patterns of NECTIN2, we conducted multiplex immunohistochemistry. This analysis demonstrated that NECTIN2 was primarily localized around epithelial cells within tumor regions, where it encircled CD8 T cells (Fig. 7G and Table S3). These findings were consistent with our results from single-cell RNA-seq and spatial transcriptomic analyses, substantiating the expression characteristics of NECTIN2 across multiple experimental platforms.

### The properties of TR CD8 + T cells’ TCR repertoire

**Fig. 8:**
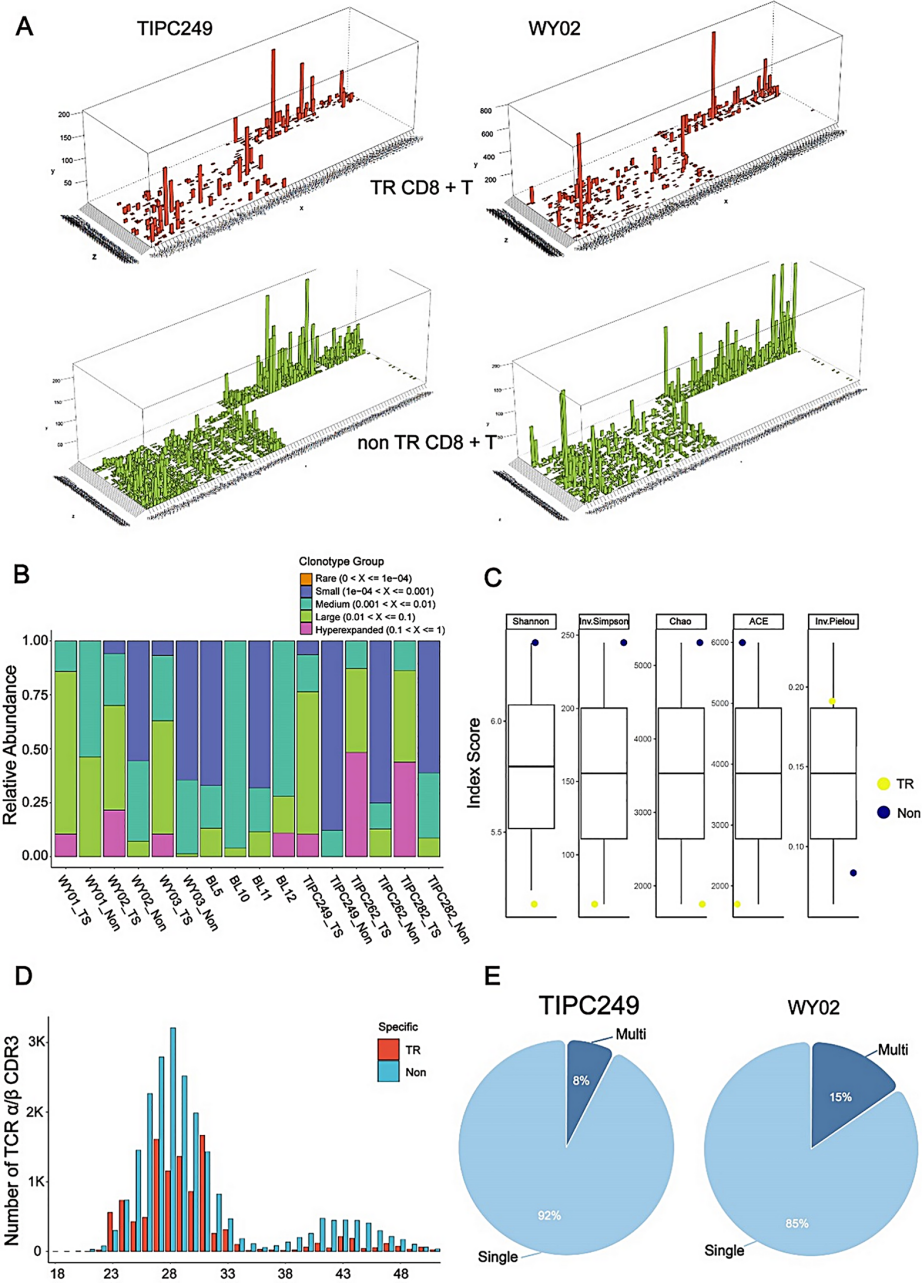
The properties of TR CD8 + T cells’ TCR repertoire. (**A**) The three-dimensional graphic images showing clonal architecture of validated patient TIPC249 and DL model predicted patient WY02. The upper image represents the clonal architecture of TR CD8 + T cells, while the lower image represents that of non-TR CD8 + T cells. (**B**) Cluster plot showing the relative abundance of CD8 + T cell clonotypes among pancreatic cancer patients (WY01/02/03, TIPC249/262/282) and pancreatitis patients (BL5/10/11/12). (**C**) Box plot showing the TCR diversity index scores of TR and non-TR CD8 + T cells. (**D**) Length distribution of the TCR αβ CDR3 chains in TR and non-TR CD8 + T cells. Data were obtained via scTCR and presented as the number of clones. (**E**) Pie chart representing the proportion of single- and multi-TCR αβ combination in validated patient TIPC249 and DL model predicted patient WY02

Subsequently, we utilized scTCR analysis to examine the characteristics of the TCR repertoire in TR CD8 + T cells in pancreatic cancer. In patients TIPC249 and TIPC262, we identified hyperexpanded clonotypes that were present in both TR and non-TR CD8 + T cells. However, the oligoclonal characteristics of TR T cells in patients WY02 and WY03 were significantly more pronounced than those of non-TR T cells (Fig. 8A and Figure S4A). This finding demonstrates that TR CD8 + T cells identification cannot rely solely on the oligoclonal nature of the TCRs. This observation is further supported by the analysis of the CD8 TCR repertoire in tissues of patients with pancreatitis, where patients with pancreatitis (BL11 and BL12) exhibited highly clonally expanded T cells in their lesions (Figure S4B). Additionally, we explored the properties of the TR CD8 + T cells’ TCR repertoire. The relative abundance of hyperexpanded clonotypes was significantly enriched in TR CD8 + TILs, whereas non-TR or pancreatitis CD8 + T cells rarely exhibited hyperexpanded clonotypes (Fig. 8B). To determine TCR diversity, we employed several commonly used indices. The analysis of clonal diversity showed that TR CD8 + TILs had a low diversity index score and a high enrichment score (Fig. 8C), indicating the concentrated and hyperexpanded clonal characteristics of TR CD8 + TILs.

We further calculated the length distribution of the TCR αβ chains, and we observed a Gaussian CDR3 length distribution pattern in non-TR CD8 + TILs. The lengths ranged from 23 to 33 amino acids, with 28 amino acids being the most common. In contrast, TR CD8 + TILs exhibited an unbalanced and concentrated distribution in the CDR3 repertoire (Fig. 8D). Interestingly, TR CD8 + TILs also included TCR αβ chains longer than 35 amino acids (Figs. 8D, E), suggesting the presence of CDR3s with multiple TCR αβ combinations that together may facilitate tumor recognition.

### The fraction of TR CD8 + T cells could be used as a potential biomarker for neoadjuvant treatment with immunotherapy

**Fig. 9:**
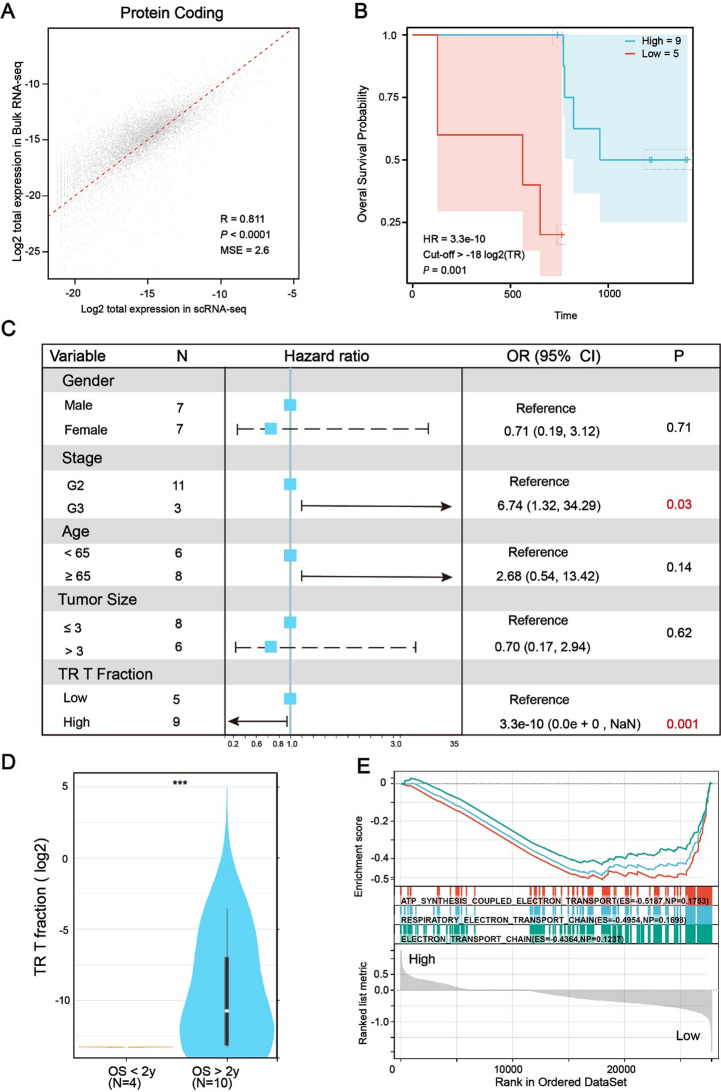
The fraction of TR CD8^+^ TILs could be used as a potential biomarker for neoadjuvant treatment with immunotherapy in human pancreatic cancer. (**A**) Protein coding genes are the most concordant group between scRNA-seq and bulk RNA-seq. MSE: mean squared error. (**B**) Curve for overall survival is shown for high and low fraction of TR CD8 + T cells in the neoadjuvant immunotherapy cohort. (logrank test, HR = 3.3e-10, *P* = 0.001). HR: Hazard Ratio. (**C**) Univariate Cox regression analysis of clinical variables as prognostic markers for neoadjuvant immunotherapy. Clinical variables include gender, stage, age, tumor size, and the fraction of TR CD8 + T cells. The TR T fraction is identified as the best prognostic marker for immunotherapy, with a highly significant p-value of 0.001. Variables with a p-value of less than 0.05 are highlighted in red. OR: Odds Ratio. (**D**) The fraction of TR CD8 + T cells was compared between OS > 2 years and OS < 2 years cohorts by non-parametric test. Data shown as the median ± SD. (Rank sum test, *** *P* < 0.001). OS: Overall Survival. (**E**) Enrichment plots showed down-regulation of mitochondrial respiratory chain pathways in sorted CD8 + TIL cells in the high fraction of TR CD8 + T cells versus low fraction of TR CD8 + T cells. ES: enrichment score; NP: Nominal P value

Following neoadjuvant therapy, the clinical prognosis of patients with pancreatic cancer varies. As we previously noted, there is substantial variation in the abundance of TR CD8 + T cells among different patients (Fig. 3G). This led us to hypothesize that TR CD8 + T cells could serve as prognostic biomarkers for immunotherapeutic outcomes in pancreatic cancer. To test this hypothesis, we performed bulk RNA sequencing on CD8 + TILs isolated from post-treatment, surgically resected PDAC in 7 patients treated with a granulocyte-macrophage colony-stimulating factor-secreting allogeneic PDAC vaccine (GVAX) and 7 patients treated with a combination of GVAX and nivolumab (Table S4. Clinical trial number NCT02451982) [46]. Using BayesPrism [40], a Bayesian statistical model, we inferred cell type composition and gene expression profiles from TR CD8 + T cells, utilizing scRNA-seq reference data as prior information. Protein-coding genes were found to be strongly correlated between the two assays (Fig. 9A, Pearson correlation *R* = 0.811, *P* < 0.0001). Patients with a high proportion of TR CD8 + T cells exhibited better clinical outcomes and longer survival times (Fig. 2B and D). Furthermore, the proportion of TR CD8 + T cells proved to be a more reliable prognostic indicator for neoadjuvant immunotherapy than traditional clinical features (Fig. 9C). To confirm the molecular pathways that were downregulated in TR CD8 + T cells based on scRNA-seq data, we conducted GSEA on bulk RNA-seq data from patients with high or low TR T cell fractions. The GSEA results were consistent with the downregulation of electron transport-related pathways in patients with a high fraction of TR CD8 + T cells, as indicated by Gene Ontology analysis (Fig. 9E).

## Discussion

CD8 + TILs in pancreatic cancer tissues play an important role in anti-tumor immune responses, and TR CD8 + TILs have a direct impact on cancer patients’ clinical prognoses. Identifying TR CD8 + TILs allows for a better understanding of the immune landscape features of TR TCRs, which can be used to develop potential targets for TCR-T cell therapy. In this work, we developed and validated a comprehensive and precise process for identifying TR CD8 + TILs using DL algorithms. TCR-T targeting and killing of tumor organoid models directly validated the tumor-specific recognition capability of the TCRs identified by our DL approach. The combination of multiple TR TCRs demonstrated a superior tumor-killing effect compared to that of individual TCRs. Through a comparative analysis with existing algorithms, we found that our proposed approach exhibits a significant advantage in identifying tumor-reactive (TR) T cells in pancreatic cancer, outperforming current methods in both sensitivity and specificity (Table S7). This superiority can be attributed to the following key factors:

1. Some existing algorithms do not distinguish between different cancer types, neglecting the biological heterogeneity of T cells across tissue microenvironments, which compromises identification accuracy;
2. Our model not only incorporates the gene expression signatures of TR cells but also accounts for the preferential distribution of T cell subtypes, significantly enhancing predictive accuracy;
3. We have addressed batch effects across different single-cell sequencing experiments, ensuring the robustness and generalizability of the algorithm in independent validation datasets;
4. To further validate our algorithm, we employed a T cell-mediated organoid cytotoxicity model, confirming its accuracy and reliability under physiologically relevant conditions.

The emergence of DL approach has enabled a more in-depth understanding of the biological characteristics of TR CD8 + T cells. We found that TR CD8 + T cells are primarily found in exhausted T cells but also have a significant presence in Tem cell subpopulations. Prior research has determined that TR T cells are predominantly concentrated in the exhausted T cell subtype [5, 47]. This disparity may be attributed to the fact that traditional identification methods require TILs expansion in vitro, resulting in TR CD8 + T cells biased toward an exhausted phenotype [8]. Using clonal information from scTCR sequencing, we identified GZMK memory subtype T cells that specifically recognized tumor PDX models, indicating that our identification method can detect TR CD8 + T cells among non-exhaustive T cells. Furthermore, the proportion of TR CD8 + T cells within the overall CD8 + TILs varies significantly among different patients. And the fraction of TR CD8 + T cells was significantly and positively related to patients’ clinical benefit and survival rate. the presence of TR CD8 + T cells may serve as a surrogate biomarker for effective immune activation following therapy. Future prospective studies with longitudinal sampling before, during, and after immunotherapy will be required to validate the functional contribution of TR cells to treatment response.

T cell activation requires substantial energy, which is primarily supplied through mitochondrial oxidative phosphorylation (OXPHOS). The electron transport chain (ETC) plays a central role in OXPHOS by coupling electron transfer with proton pumping to generate ATP, thereby fulfilling the metabolic demands of activated T cells [48, 49]. Our transcriptomic analysis revealed a sustained downregulation of mitochondrial respiratory chain genes across various TR CD8⁺ T cell subsets, with a pronounced suppression of Complex I–associated genes. This suggests a widespread impairment of mitochondrial function in TR T cells. Deficiency in Complex I leads to reduced ATP production, redox imbalance, and T cell dysfunction. Notably, CD8⁺ T cells from Complex I–deficient mice exhibit a greater propensity toward exhaustion and diminished inflammatory cytokine production [50]. Multiple factors within the TME contribute to mitochondrial dysfunction in T cells. These include the high glucose uptake by tumor cells (the Warburg effect), which limits glucose availability for T cell glycolysis [51]; accumulation of lactate, which inhibits mitochondrial complex activity, particularly Complex I [52]; elevated reactive oxygen species (ROS), which can directly damage mitochondrial DNA, membrane potential, and ETC complexes; and tumor cell–mediated mitochondrial hijacking through tunneling nanotubes [53]. Our data further suggest that the FOS transcription factor regulatory network plays a critical role in the transcriptional control of mitochondrial genes in T cells. Persistent stimulation of T cells may disrupt FOS-mediated transcriptional regulation, leading to impaired mitochondrial respiratory chain function—an observation that warrants further investigation.

Cell communication analysis revealed that, in the TME, TR CD8 + T cells’ TIGIT signaling pathway frequently interact with those of other cell types, especially epithelial cells in tumor tissues. Previous studies in pancreatic cancer have focused on the TIGIT ligand CD155 [54]. Our study showed that NECTIN2 is another important ligand of TIGIT in pancreatic cancer. The interaction between TIGIT and NECTIN2 has been demonstrated to play a critical immune checkpoint regulatory role within the tumor immune microenvironment of breast carcinoma and neuroblastoma [55, 56]. Analysis of the TCR repertoire in TR CD8 + T cells revealed that relying solely on the oligoclonal nature of the TCR is insufficient for accurately identifying TR T cells. Because highly expanded T cell clones are also present within non-TR CD8 + T cell populations. This finding raises concerns about the reliability of a recently published method that relies solely on TCR oligoclonality to identify tumor-reactive T cells, highlighting potential limitations in its approach [57]. More importantly, whether TR CD8 + T cells were validated through the PDX model or identified by DL models, we observed a significant proportion of TR CD8 + T cells exhibiting multiple TCR α/β pairings, exceeding the systematic error of the 10x sequencing platform (6%). This may be caused partly by naturally occurring multiplets of α/β-chain (4%) due to the incomplete gene restriction of the thymocyte during negative selection [58, 59]. This type of T cell, which includes multiple TCR receptors, statistically increases the likelihood of recognizing tumors.

While our findings are robust across multiple modalities, the functional and spatial analyses are derived from a limited cohort, and future studies involving larger, multi-center patient cohorts are necessary to confirm the generalizability of these observations. Nevertheless, the DL approach does not require neoantigen screening or in vitro expansion culturing, which reduces the cost and time required to identify TR CD8 + T cells in pancreatic cancer. Therefore, DL-based algorithms for identifying TR T cells will enable researchers and clinicians to identify TR CD8 + T cells in pancreatic cancer more rapidly. The strategy for identifying TR TILs in pancreatic cancer might be applicable to other tumor types.

## Conclusions

Overall, we have developed a rapid, sensitive, and specific deep learning-based method for identifying TR CD8 + TILs in pancreatic cancer. This approach provides valuable insights into the biological functions of TR CD8 + TILs and holds significant potential for advancing the development of TCR-T cell immunotherapy.

## Supplementary Information

Below is the link to the electronic supplementary material.

Supplementary Material 1: Table S1. Metadata for cohorts used in this study

Supplementary Material 2: Table S2. The blacklist genes

Supplementary Material 3: Table S3. Quantitative summary of multiplexed immunofluorescence (mIF) analysis in tumor and non-tumor regions

Supplementary Material 4: Table S4. Clinical information of the neoadjuvant immunotherapy cohort for pancreatic cancer

Supplementary Material 5: Table S5. Clinical characteristics of patients in this study

Supplementary Material 6: Table S6. DEGs of T cell subtypes

Supplementary Material 7: Table S7. Evaluation of different algorithms

Supplementary Material 8

Supplementary Material 9

## Data Availability

The single-cell datasets generated during this investigation are available in the zenodo database https://zenodo.org/records/14213503). The source of the original data is provided in this paper. The DL model and all analysis process codes have been uploaded to the GitHub website (https://github.com/shizhiwen1990/TR).
