# Supplementary material for: Functional tumor-reactive CD8 + T cells in pancreatic cancer
Source: J Exp Clin Cancer Res. 2025 Aug 25;44:253. doi: 10.1186/s13046-025-03517-1 (PMC12379552; doi:10.1186/s13046-025-03517-1)
Supplement: Supplementary file 9 — Supplementary Material 9 [file 13046_2025_3517_MOESM9_ESM.docx]

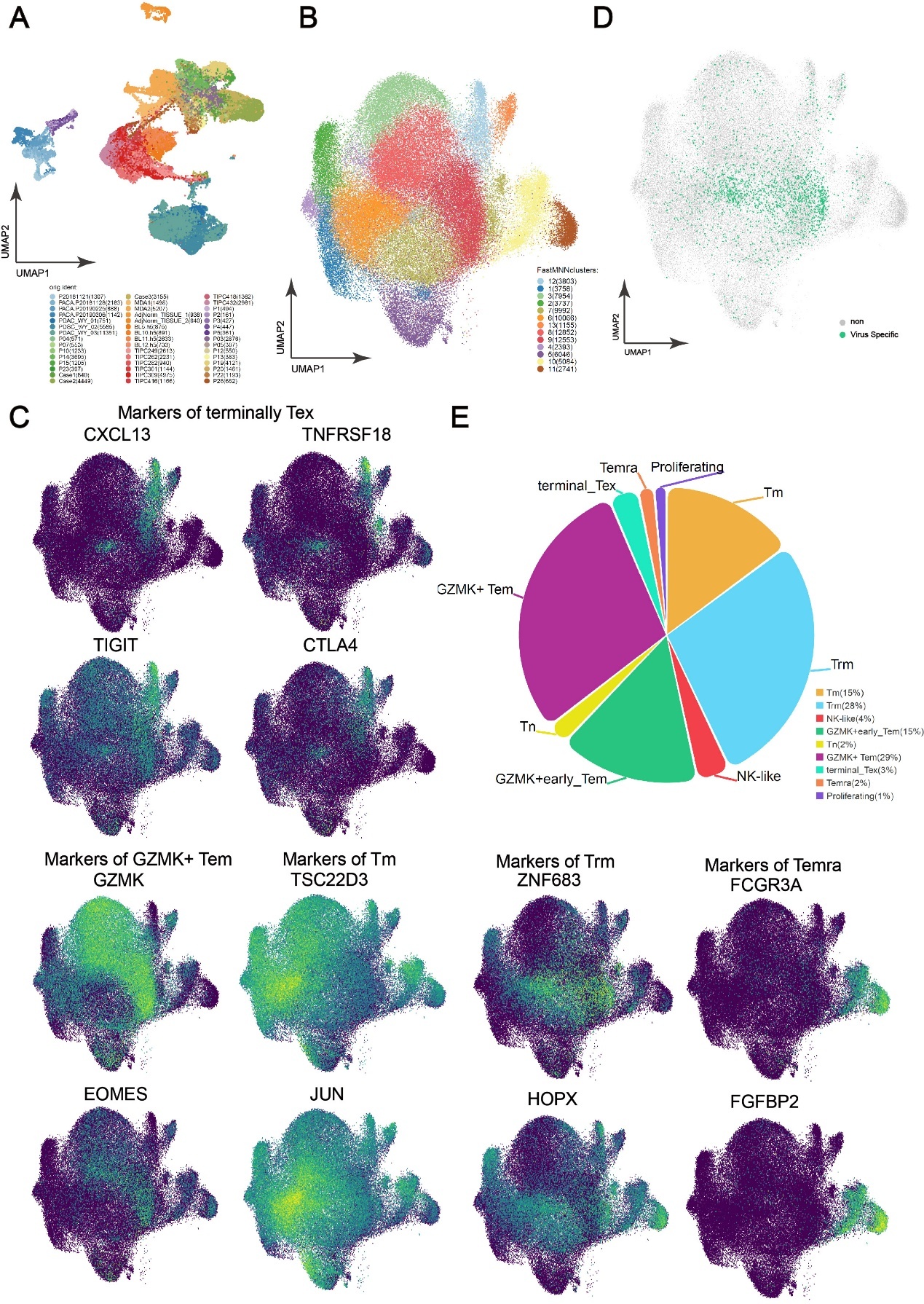


**Figure S1**. (A) UMAP clustering plot of different samples before batch effect correction. (B) UMAP plot of CD8+ TILs from 41 pancreatic cancer patients and 4 pancreatitis patients (BL5/10/11/12). (C) Feature plots displaying classical marker genes used for the annotation of these subclusters. (D) UMAP plot of all virus-specific CD8+ TILs from all patients. (E) Pie chart representing the proportion of virus-specific CD8+ TILs in different T cell subtypes.


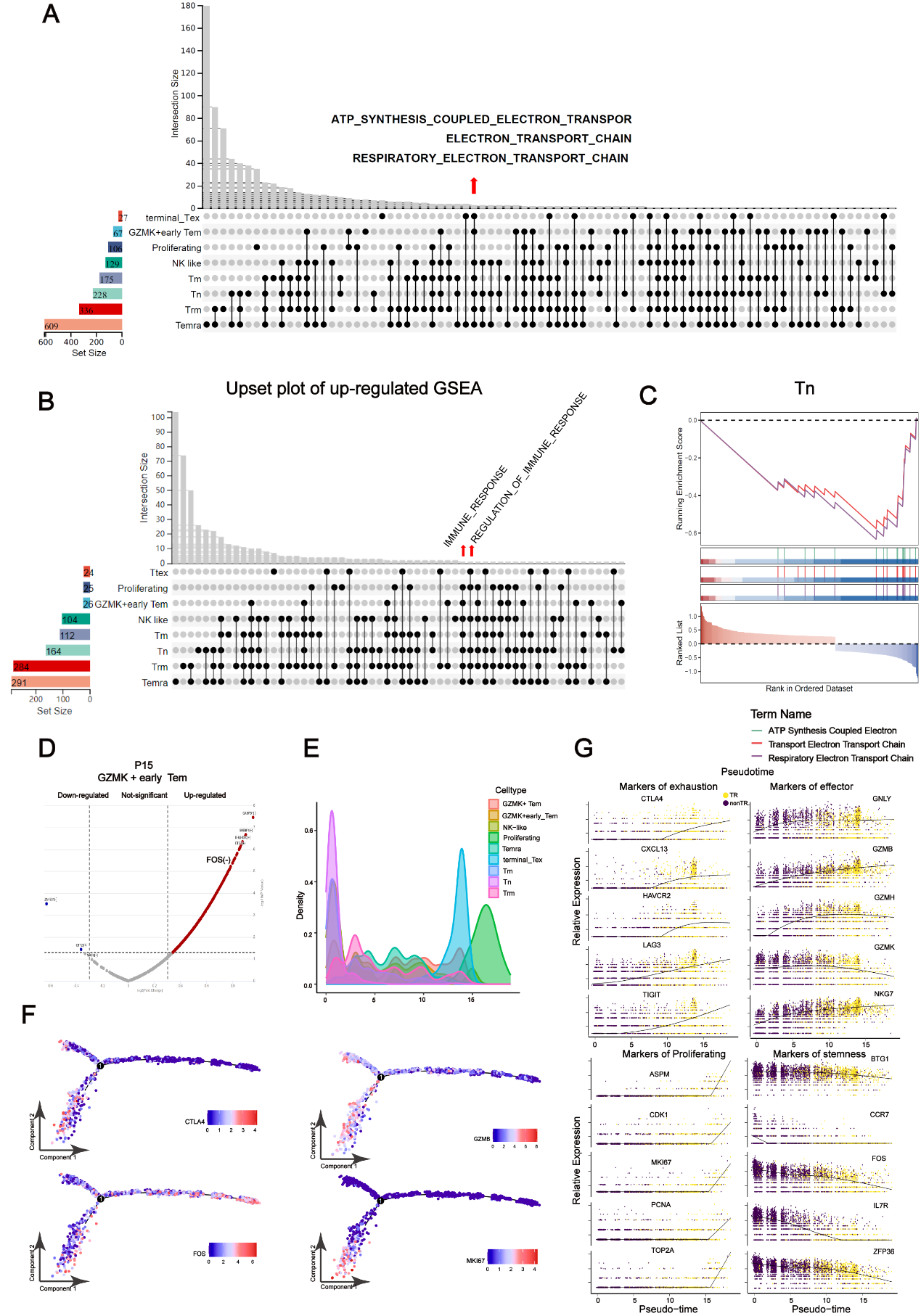


**Figure S2**. (A) Upset plot showing the overlap of total GO pathways between the indicated comparisons of the different subtypes of TR CD8+ T cells. (B) Upset plot showing the overlap of up-regulated GO pathways between the indicated comparisons of the different subtypes of TR CD8+ T cells. (C) Enrichment plot for the set of DEGs in the transcriptome of TR CD8+ T cells versus non-TR T cells in Tn subtype by GSEA. (D) Volcano plot showing the significantly downregulated FOS (+) regulon in TR CD8+ T cells of patient P15. (E) Density plot illustrating the distribution of various T cell types along pseudotime. (F) Gene expression dynamics along a developmental trajectory, represented in a pseudotemporal space defined by Component 1 and Component 2 axes. Each subplot corresponds to a specific gene: CTLA4, FOS, GZMB, and MKI67. (G) Two-dimensional plots showing the dynamic expression of selected genes along the pseudo-time, colored by TR or nonTR T cells.


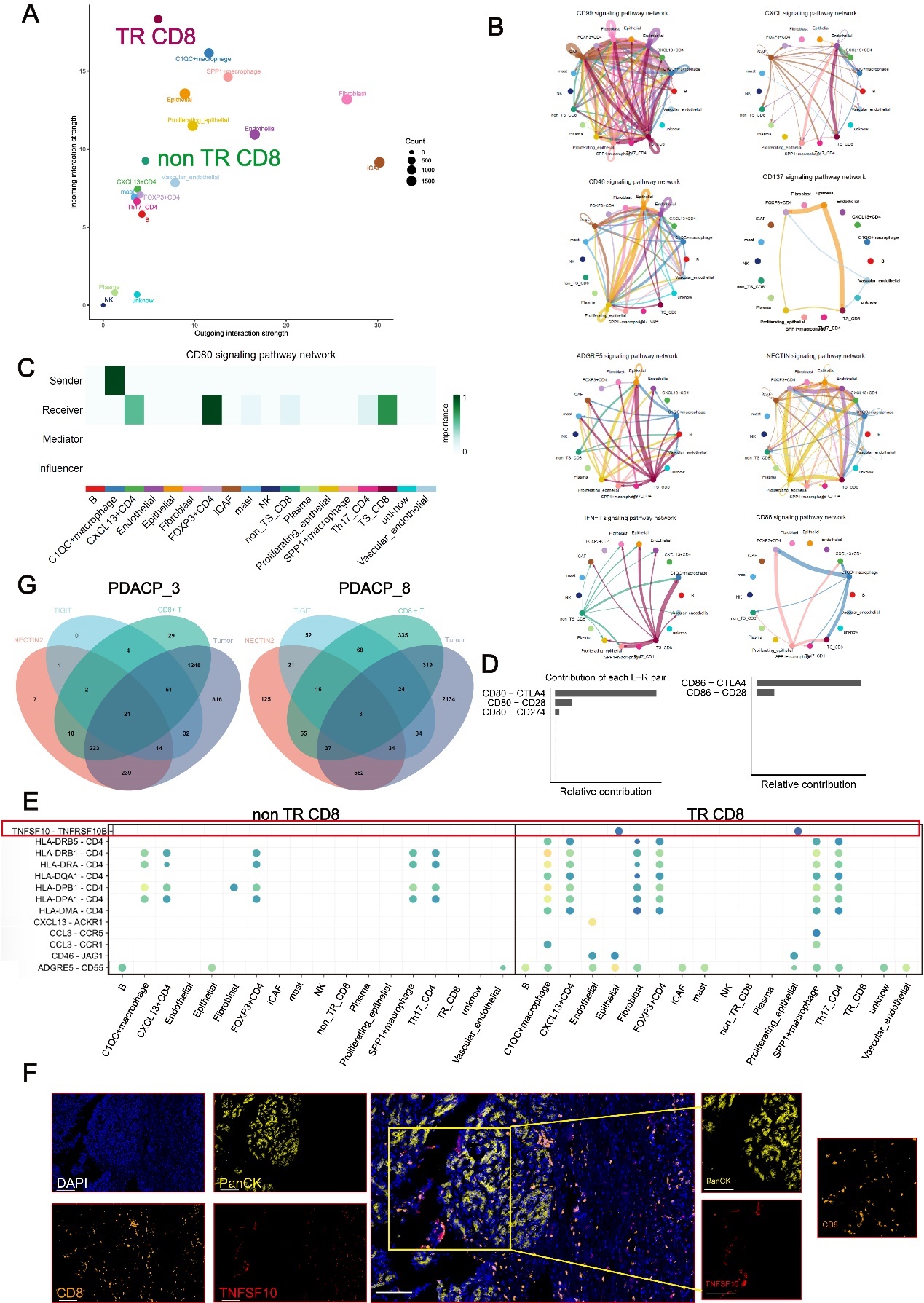


**Figure S3**. (A) The scatter plot depicts the incoming and outgoing interaction strength of the 18 annotated cell subtypes in all signals. (B) Intercellular communication networks showing the strength of cell subtypes in twelve distinct signaling pathway networks. (C) The heatmap plot illustrating the significantly different incoming and outgoing CD80 pathway between TR and non-TR CD8+ TILs. (D) The ligand–receptor analysis revealing activated interactomes between TR CD8+ TILs and other cell subtypes, comparing to non-TR CD8+ T cells and other cell subtypes. (E) Dot plot showing TR CD8 cell-specific receptor-ligand signaling. (F) Tumor sections from patients with pancreatic cancer stained with anti-PanCK (yellow), anti-TNFSF10 (red), anti-CD8(orange) and DAPI. (G) Venn diagram illustrating the spatial overlap of TIGIT-positive, NECTIN2-positive, Tumor-positive, and CD8-positive spots in the spatial transcriptomic analysis of the two patients. For patient PDACP_3, 62.4% of TIGIT-positive spots colocalized with CD8-positive spots, 22.4% overlapped with NECTIN2-positive spots, and 56.42% of Tumor-positive spots colocalized with CD8-positive spots. For patient PDACP_8, 55.94% of TIGIT-positive spots overlapped with CD8-positive spots, 37.76% colocalized with NECTIN2-positive spots, and 71.64% of Tumor-positive spots were shared with CD8-positive spots.


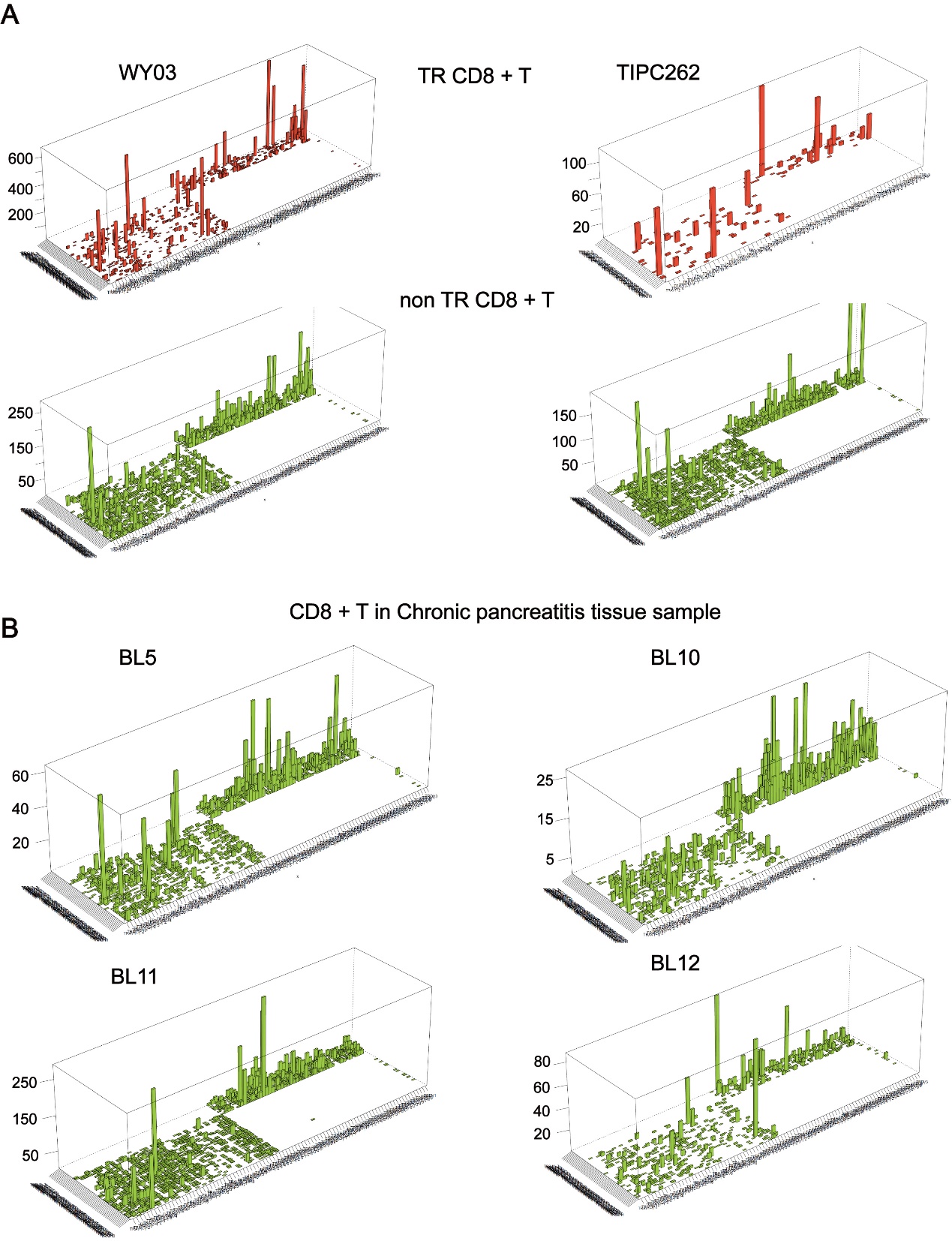


**Figure S4**. (A) Three-dimensional graphic images showing the clonal architecture of validated patient TIPC262 and DL model-predicted patient WY03. Upper image represented the TR CD+ T cells clonal architecture and lower image represented the non-TR CD+ T cells clonal architecture. (B) The three-dimensional graphic images showing clonal architecture of 4 chronic pancreatitis tissue sample.
